# A Nutrient Combination that Can Affect Synapse Formation

**DOI:** 10.3390/nu6041701

**Published:** 2014-04-23

**Authors:** Richard J. Wurtman

**Affiliations:** Department of Brain and Cognitive Sciences, Massachusetts Institute of Technology, 77 Mass Ave., 46-5009, Cambridge, MA 02139, USA; E-Mail: dick@mit.edu

**Keywords:** uridine, docosahexaenoic acid (DHA), choline, synapse, dendritic spine, neurite, phosphatidylcholine (PC), uridine monophosphate (UMP), Alzheimer’s disease, neurodegenerative diseases

## Abstract

Brain neurons form synapses throughout the life span. This process is initiated by neuronal depolarization, however the numbers of synapses thus formed depend on brain levels of three key nutrients—uridine, the omega-3 fatty acid DHA, and choline. Given together, these nutrients accelerate formation of synaptic membrane, the major component of synapses. In infants, when synaptogenesis is maximal, relatively large amounts of all three nutrients are provided in bioavailable forms (e.g., uridine in the UMP of mothers’ milk and infant formulas). However, in adults the uridine in foods, mostly present at RNA, is not bioavailable, and no food has ever been compelling demonstrated to elevate plasma uridine levels. Moreover, the quantities of DHA and choline in regular foods can be insufficient for raising their blood levels enough to promote optimal synaptogenesis. In Alzheimer’s disease (AD) the need for extra quantities of the three nutrients is enhanced, both because their basal plasma levels may be subnormal (reflecting impaired hepatic synthesis), and because especially high brain levels are needed for correcting the disease-related deficiencies in synaptic membrane and synapses.

## 1. Introduction

The recognition that nutrient intake can affect the rate at which the brain makes new synapses arose largely from basic-science studies performed in MIT’s Department of Brain and Cognitive Sciences between 1999 and 2012 [1,2,3,4]. As described below, these studies showed that when three nutrients—uridine (as its monophosphate, UMP); the omega-3 fatty acid DHA (or EPA, which is equally effective [4]); and choline—are consumed together, brain cells produce more of the specialized membranes [1] used for forming dendritic spines [2,3], the immediate anatomic precursor of synapses. The nutrients also improve cognitive test scores in both impaired and normal experimental animals [2,4]. Based on these findings, and on the consensus that a deficiency in synapses underlies the memory disorder seen in early Alzheimer’s disease [5], a mixture containing the three nutrients plus others that enhance their efficacy, SOUVENAID^®^ was formulated and tested, and found to improve memory scores [6] and the connectivities between brain regions [7] among patients with early Alzheimer’s Disease.

The ability of the brain to make more phosphatidylcholine (PC), and related membrane constituents when presented with additional uridine, DHA, and choline, requires that the enzymes which catalyze the steps in PC synthesis have a special biochemical property: Each enzyme must exhibit unusually poor affinity for its substrate, so that, at the uridine, DHA, and choline concentrations normally present in brain cells, many enzyme molecules will not be attached to their substrate and, thus, will not be able to act on it. However, when additional substrate is provided—by administering the nutrients to raise their blood levels—more enzyme molecules become engaged, and more of their product is formed [2]. We had first observed a similar “precursor-dependence” many years earlier involving the conversion of a different nutrient, the amino acid tryptophan, to serotonin, a neurotransmitter [8]: Giving animals tryptophan rapidly increased the formation and release of brain serotonin. Similar precursor-dependence was subsequently shown to characterize numerous other neurotransmitters [9].

The concentrations of uridine, DHA, and choline required for engaging just half of the molecules of each enzyme needed for converting choline to PC (*i.e.*, each enzyme’s Michaelis-Menten constant [Km]) were found to be very high in relation to their actual brain concentrations. Hence, administering each of the nutrients does in fact increase the production, and, ultimately, the levels of brain PC [1]. Moreover, the effect of giving uridine, DHA, and choline together is additive or greater. An additional biochemical mechanism activated by the uridine in SOUVENAID^®^ enables the brain also to increase production of the special proteins found in synaptic membranes. It involves the activation of P2Y receptors, a particular family of brain receptors that can affect neuronal differentiation and synaptic protein synthesis [10]. P2Y receptor activation is also involved in the processes that shape the newly-formed membrane into neurites, dendritic spines, and synapse [10].

## 2. Control of Synaptogenesis

Most of the communications that take place between a brain neuron that transmits a signal and one that receives that signal occur at synapses. These are highly specialized structures, which include a presynaptic terminal budding from the transmitting neuron’s axon; the postsynaptic membrane, usually on one of the receiving neuron’s dendrites; and the space between the neurons—the synaptic cleft. Presynaptic terminals make and store the neuron’s neurotransmitter, and release some of it into the synaptic cleft when the neuron is depolarized. The postsynaptic membranes contain receptors to which the neurotransmitter can then attach, as well as other protein molecules which mediate the functional consequences of receptor activation. Pre- and post-synaptic membranes are composed principally of phosphatides like PC; other phospholipids and cholesterol; and particular proteins, many of which are confined to synaptic structures.

The postsynaptic membranes on which glutamate, the most widely-used excitatory brain neurotransmitter, acts contain large numbers of dendritic spines, as well as postsynaptic densities (PSD) which house various proteins that mediate most of the functional consequences of receptor activation. For example, some of these proteins open or close “pores” in the membranes, thereby allowing specific ions which affect the cell’s voltage to pass in or out; others activate enzymes that synthesize second-messenger compounds (like cyclic-AMP or diacylglycerol), which affect gene expression and the overall metabolism of the post-synaptic neuron.

The formation of a new synapse (by, for example, hippocampal neurons that use glutamate as their neurotransmitter) can be initiated by the coming together of what will become a presynaptic terminal and a dendritic spine. Thus the availability of dendritic spines is an important factor controlling the rate of synaptogenesis. This number can be increased by various treatments (e.g., by giving the hormone ghrelin—which also enhances memory performance and long-term potentiation, a form of learning), or decreased by Alzheimer’s and other dementia-producing diseases. The sparsity of dendritic spines in AD brain [11] probably causes a decrease in the formation of new synapses. This may partially explain the decreased number of cortical synapses and the consequent memory impairment observed early in the course of the disease.

Although most brain synapses are formed prenatally or during early postnatal development, each survives for only days to months, and must be renewed periodically throughout the individual’s life span. This continuing necessity is probably a major factor underlying the brain’s plasticity and the individual’s ability to learn, since it allows specific, newly-formed synapses to be associated with newly-learned material. Early in development, most synaptogenesis occurs in the absence of neuronal depolarization and neurotransmitter release. In adulthood, however, the rate at which new synapses form, and the ways their connections become configured, are largely governed by neuronal activity. This allows very active synapses to facilitate the formation of additional functionally-related synapses. However, the *magnitude* of the increase in synaptogenesis among active neurons is apparently modulated by nutrient availability, specifically of uridine, DHA, and choline [1].

## 3. Biosynthesis of Membrane Phosphatides, Synaptic Proteins, Neurites, and Dendritic Spines: Effects of Uridine, Dha, and Choline

### 3.1. Membrane Phosphatides

All cells utilize DHA and other fatty acids (e.g., EPA); uridine; and choline to form the phosphatide compounds that constitute the major components of their membranes. PC, the most abundant phosphatide in brain, is synthesized from these precursor-nutrients by a set of enzymes that comprise the CDP-choline cycle (or “*Kennedy Cycle*”). This biochemical pathway also generates a related membrane constituent, the phosphatide phosphatidylethanolamine (PE). Phosphatidylserine (PS), the third major membrane phosphatide, is produced by exchanging a serine molecule for the choline in PC or the ethanolamine in PE. Sphingomyelin, the other major choline-containing brain constituent, is formed from PC. Thus, all of the principal lipid components of synaptic membranes are affected by the rate at which PC is being formed. In addition, since each of the reactions needed to convert choline, uridine, and DHA to PC is catalyzed by a low-affinity enzyme, blood levels of the three nutrient-precursors can determine not only PC’s rate of synthesis but also the rates at which almost all of the brain’s membrane lipids are produced. When all three of the nutrients are provided concurrently the resulting increase in PC production is greater than the sum of the increases produced by giving each separately [1,2]. This probably occurs because if just one of the nutrient-precursors were to be provided, the concentrations of the other two would continue to be limiting.

The CDP-choline cycle involves three sequential enzymatic reactions [2]: In the first, catalyzed by choline kinase (CK), a phosphate group is transferred from ATP to the hydroxyl oxygen of choline, yielding phosphocholine. The second reaction, catalyzed by CTP:phosphocholine cytidylyltransferase (CT), transfers cytidine-5′-monophosphate (CMP), a portion of the high-energy CTP molecule, to the phosphocholine, yielding cytidine-5′-diphosphocholine (also known as CDP-choline, or as citicoline). (As discussed below, most of the CTP that the human brain uses for PC synthesis comes from circulating uridine, there being little or no free cytidine in human blood) [12]. The third and last reaction, catalyzed by the enzyme CDP-choline:1,2,diarylglycerol cholinephosphotransferase (CPT), transfers the choline moiety from CDP-choline to the free hydroxyl group of diacylglycerol (DAG), yielding the PC. As described below, the brain obtains all three of these PC precursors mostly or entirely from the blood stream.

Thus, administering choline increases brain phosphocholine levels in rats and humans because CK’s Km for choline (2.6 mM) is much higher than usual brain choline levels (30–60 µM) [2]. Similarly, administering uridine increases brain levels of UTP and then CTP [2], and giving DHA increases the concentration of DAG molecules likely to be used for production of PC (because one of their two fatty acids happens to be DHA, an omega-3 compound).

### 3.2. Proteins Localized in Pre- and Post-Synaptic Membranes

Administration together of DHA, uridine (as UMP) and choline also increases the levels of proteins that are localized within presynaptic and postsynaptic membranes (for example, synapsin-1P; syntaxin-3; PSD-95), but not of other proteins, like beta-tubulin, which are found all over the brain [1,2,3]. The increase in synaptic proteins probably results from the additional action of uridine described above, *i.e.*, its ability (shared with uridine-containing nucleotides like UTP) to activate P2Y receptors in the brain [10]. Nucleotides like UTP activate a variety of receptor subtypes that stimulate either ion fluxes (P2X) or intracellular metabolic processes (P2Y). The P2Y receptors in brain (P2Y2; P2Y4; P2Y6) tend to be specific for uridine-containing compounds. Their activation increases intracellular concentrations of the second-messengers DAG, inositol triphosphate, and calcium.

### 3.3. Neurite Outgrowth

Activation of P2Y receptors by uridine or UTP also affects the outgrowth, from neuron-derived cells, of neurites [10] (which, like dendritic spines, can be precursors for synapses, and are principally composed of membranes). P2Y activation can also stimulate neuronal differentiation; the branching of the neurites; protein synthesis; expression of neurofilament proteins present in neurites; neurotransmitter release; and the enhancement of long-term potentiation. All of these effects can be blocked by drugs that antagonize P2Y receptors. The increase in neurites among cells exposed to uridine or UTP [10] provided the first demonstration that phosphatide precursors could alter cellular anatomy, as well as biochemical indices.

P2Y2 receptors in patients with AD apparently are deficient in the parietal cortex [13]—a brain region important for memory. This suggests that increasing brain levels of the receptor ligand UTP—by administering the UMP in SOUVENAID^®^—may partially compensate for the P2Y receptor deficiency.

Interestingly, uridine is not unique in being able to regulate cell differentiation and metabolism by two distinct mechanisms, acting both as a receptor agonist and as a bulk precursor of the CTP needed for phosphatide synthesis. Diacylglycerol also acts in two ways, both as a potent “second messenger” that activates a number of enzymes and other proteins, and also, like uridine, as a bulk precursor in phosphatide synthesis.

### 3.4. Incorporation of Synaptic Membrane into Dendritic Spines

The numbers of dendritic spines in particular brain regions are highly correlated with the numbers of synapses [3], and it has been proposed that “more than 90% of excitatory synapses occur on dendritic spines” [2]. This suggests that processes that damage the spines or, conversely, that increase spine number will cause parallel changes in synapse number. The formation of new dendritic spines in the hippocampus can be initiated by synaptic inputs that depolarize CA1 pyramidal neurons and cause long-term potentiation. This effect is probably mediated by enhanced calcium influx into the CA1 neurons.

The effects on hippocampal dendritic spine number of administering the omega-3 fatty acid DHA, alone or with UMP, were compared after one to four weeks of daily treatment [3]. DHA alone caused dose-related increases in spine number, as well as in membrane phosphatides and synaptic proteins; its effects were doubled if animals also received UMP. Treated animals also exhibited improvements in cognitive performance as assessed using the Morris water maze test and various other tests based on traversing mazes [4]. In contrast, administration of the omega-6 fatty acid arachidonic acid affected neither spine density nor membrane composition. Similar positive effects of giving DHA plus UMP were observed in weanlings whose mothers consumed the nutrients for 10 days prior to parturition, and for 21 days while nursing.

In summary, the simultaneous provision of DHA, uridine (as UMP) and choline increases brain phosphatides and synaptic proteins, the main constituents of synaptic membranes, as well as dendritic spines, the immediate anatomic precursor for new synapses.

## 4. Sources of Brain and Blood Uridine, Dha, and Choline

### 4.1. Blood-Brain Transport

#### 4.1.1. Uridine

Circulating uridine is carried across cellular membranes in all tissues, including the brain, by two families of transport proteins: a low-affinity, facilitated-diffusion system—which probably operates only when plasma uridine levels have been increased substantially, (e.g., after administration of a UMP source, like mothers’ milk) and a high-affinity system, which operates under physiological conditions [2], and which can maintain a concentration gradient between brain and blood. On entering brain cells uridine is phosphorylated, initially to UMP, and subsequently retained.

#### 4.1.2. DHA

DHA enters the brain, and then brain cells, both by a simple diffusion mechanism and through the action of one or more transport proteins [2]. Once inside cells it is activated by combining with -CoA, and the DHA-CoA is used for DAG. The enzyme that catalyzes DHA’s acylation has a low affinity relative to usual brain levels, so that raising blood DHA levels rapidly increases DHA’s uptake into and retention by brain cells.

#### 4.1.3. Choline

Choline molecules (but not PC nor its metabolite lyso-PC) enter the brain principally by facilitated bidirectional diffusion, mediated by a transport protein in the endothelial cells lining the brain’s capillaries [2]. The rate of choline’s entry depends on the gradient between its concentrations in the brain’s extracellular fluid (ecf) and in the blood. Once in the ecf, choline can enter cells by a ubiquitous, low-affinity transport system or, in the terminals of acetylcholine-producing neurons, by a high-affinity system.

### 4.2. Sources of Blood Uridine, DHA, and Choline

#### 4.2.1. Blood Uridine

The sources of uridine in human blood depend largely on the age of the human: In infancy, as described below, mother’s milk or infant formulas can provide substantial uridine in the form of UMP. In adulthood however, almost all of the uridine in the blood has been synthesized by the liver—unless one consumes a UMP-fortified mixture, like SOUVENAID^®^.

In humans, uridine is apparently the only pyrimidine nucleoside in the circulation [12]: plasma cytidine levels—which are high in, for example, rodents—are very low, even after administration of a cytidine source, because the cytidine is rapidly converted to uridine in the human liver. Hence, plasma uridine is the sole circulating precursor for CTP and other cytidine-containing compounds in human brain. The uridine in blood of adult humans is synthesized in the liver as UMP, and secreted as such into the circulation. In contrast, the uridine in the blood of infants can derive from the UMP in mothers’ milk or, since the 1990s, from the UMP added to infant formulas [14,15] as a “conditionally-essential nutrient” [14,15,16] thought to promote the growth of rapidly-dividing gastrointestinal and immune cells [16]. Uridine is also present in most foods consumed after infancy principally in the form of RNA. But unlike free uridine or the uridine in UMP, the uridine in RNA is not generally bioavailable [17], and no uridine-containing adult food has ever been compellingly demonstrated to raise plasma uridine levels in humans. As Gasser, *et al.* wrote in 1981 [17]: “*The liver appears to degrade essentially all incoming uridine contained in the portal drainage derived from intestinal absorption and peritoneal organs. Hepatic pools of acid-soluble nucleosides* [e.g., UMP] *formed almost completely by de novo synthesis, are responsible for the uridine compounds in plasma leaving the liver through the hepatic vein.*” It can be conjectured that one special advantage that accrues to infants by consuming mother’s milk, a source of bioavailable uridine, relates to their need for large amounts of uridine to produce sufficient synaptic membrane for the rapidly-growing brain. In addition, if AD patients with depleted cortical synapses also need to accelerate synaptic membrane synthesis, then uridine, as such or in its most bioavailable form, UMP; also becomes for them a “*conditionally essential nutrient*”.

Plasma uridine levels are apparently subnormal in patients with very mild AD [18,19]. The rates at which livers of AD patients synthesize and secrete uridine or UMP apparently have not yet been examined.

#### 4.2.2. Sources of Blood DHA

Circulating DHA also can derive both from endogenous synthesis (from alpha-linolenic acid [ALA], in the liver) and from consumption of DHA-rich foods (e.g., fatty fish). The enzymatic conversion of ALA to DHA is reportedly impaired in some patients with AD [20] because of a genetic lesion that lowers the activity of perioxisomal D-bifunctional protein, the enzyme that catalyzes the last step in this conversion. Hence DHA levels in the plasma [21], liver, and various brain regions of AD patients are reduced. Moreover the ratio in liver of the enzyme’s product—DHA—to its substrate—linolenic acid—is subnormal, and correlates with AD patients’ abnormally low scores [20] in a standard test of cognition (MMSE).

The existence of this genetic lesion, which may constitute a risk factor for AD, suggests that AD may involve more than just the brain. It also indicates that AD patients need a supplemental source of DHA both to obtain normal blood and tissue DHA levels and to promote synaptogenesis. Another metabolic factor that might affect plasma DHA levels in AD patients is the concurrent reduction in plasma levels of three vitamins [22]—B12, folate, and B6—needed for regenerating the methyl groups in methionine. This reduction impairs hepatic choline synthesis, as discussed below, and may in itself affect plasma DHA levels.

#### 4.2.3. Sources of Blood Choline

Like uridine and DHA, plasma choline can derive both from its hepatic synthesis—as PC, formed from the B-vitamin-dependent sequential methylation of PE, which can then be hydrolyzed to free choline—and from dietary sources (e.g., egg yolks, principally in the form of PC). A recommended dietary intake of choline by normal adults has been proposed as being about half a gram per day, however, many people—particularly the aged, and women in general including pregnant women—often fail to attain this level [23,24]. Moreover, basal dietary choline requirements may be significantly greater among the many individuals who have common variations in certain gene alleles for hepatic enzymes, *i.e.*, PEMT, which converts PE to PC, or for 5,10-methylenetetrahydrofolate dehydrogenase [MTHFR]) [23,24]: Premenopausal women with the allele for the less active MTHFR were found to be 15 times more susceptible than control subjects to developing organ dysfunction when placed on a low-choline diet [24].

To some extent an increased need for dietary choline—as occurs in people who, usually-unknowingly, carry such alleles, or in AD patients who require additional choline to enhance synaptic membrane synthesis—can be satisfied by providing oral choline. However when doses in excess of 500 mg are consumed many subjects develop an unacceptable “fishy” body odor, caused by choline’s degradation to trimethylamine by bacteria in the large intestine. Hence SOUVENAID^®^ also contains, besides choline, three vitamins—B12, folate, and B6—which promote endogenous choline synthesis in the liver [22].

## 5. Conclusions

Although the formation of new synapses is triggered by neuronal firing, the number of synapses that form can be modulated when three circulating nutrients, uridine, DHA, and choline are administered together. This is because cellular levels of the nutrients control the saturation of key enzymes in the synthesis of the phosphatides in synaptic membranes. One of the nutrients, uridine, also affects synaptogenesis by activating P2Y receptors in the brain.
